# Supplementary material for: Interactions between the 2C protein of foot-and-mouth disease virus and components of the viral replication machinery are mediated by endoplasmic reticulum-derived membranes
Source: J Gen Virol. 2026 May 14;107(5):002244. doi: 10.1099/jgv.0.002244 (PMC13175506; doi:10.1099/jgv.0.002244)
Supplement: Uncited Supplementary Material 1. [file jgv-107-02244-s001.pdf]

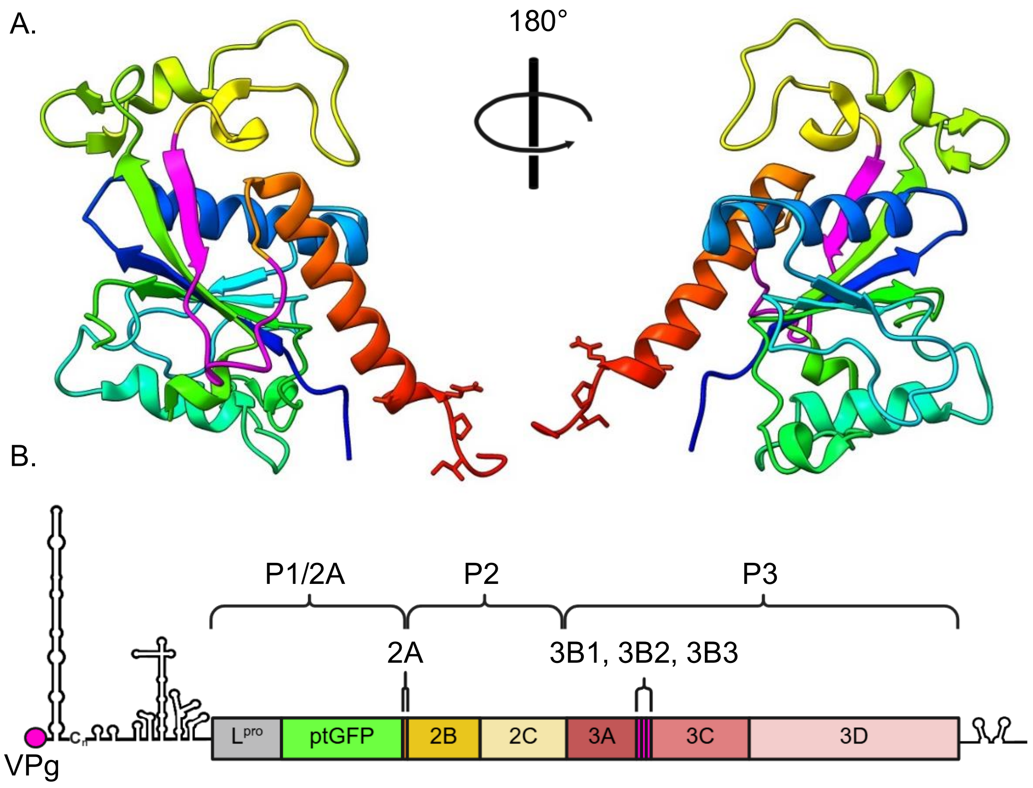

Figure S1 Mapping of peptide location within FMDV 2C structure used for generation of anti-2C antibody and FMDV replicon organisation cartoon. (A) Peptide residues (pink) used to generate novel anti-2C antibody mapped onto the crystal structure of FMDV protein residues 97-318 aa (PDB 7E6V), coloured N- to C- terminal rainbow gradient (Zhang *et al.*, 2022). (B) Cartoon representation of FMDV replicon organisation highlighting protein encoding order and untranslated region structures present.

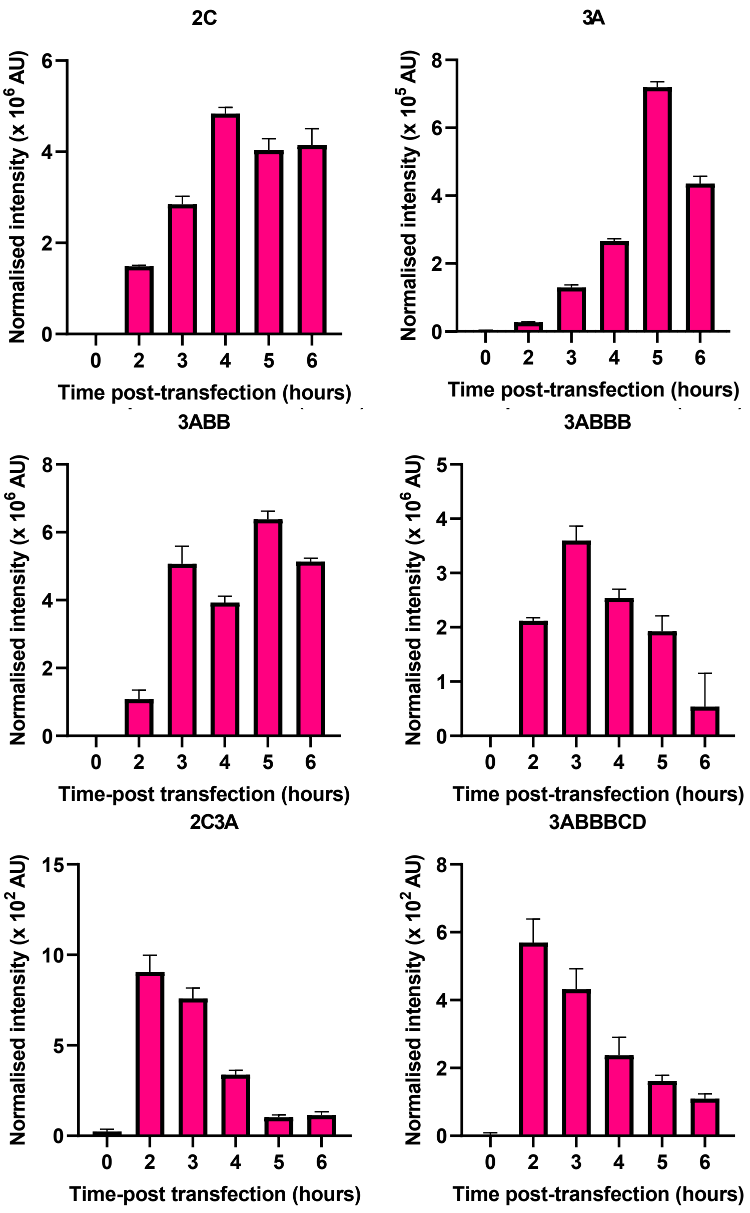

Figure S2. Densitometry of FMDV protein expression over time. Densitometry of signal corresponding to relevant molecular weight bands for FMDV precursor proteins.

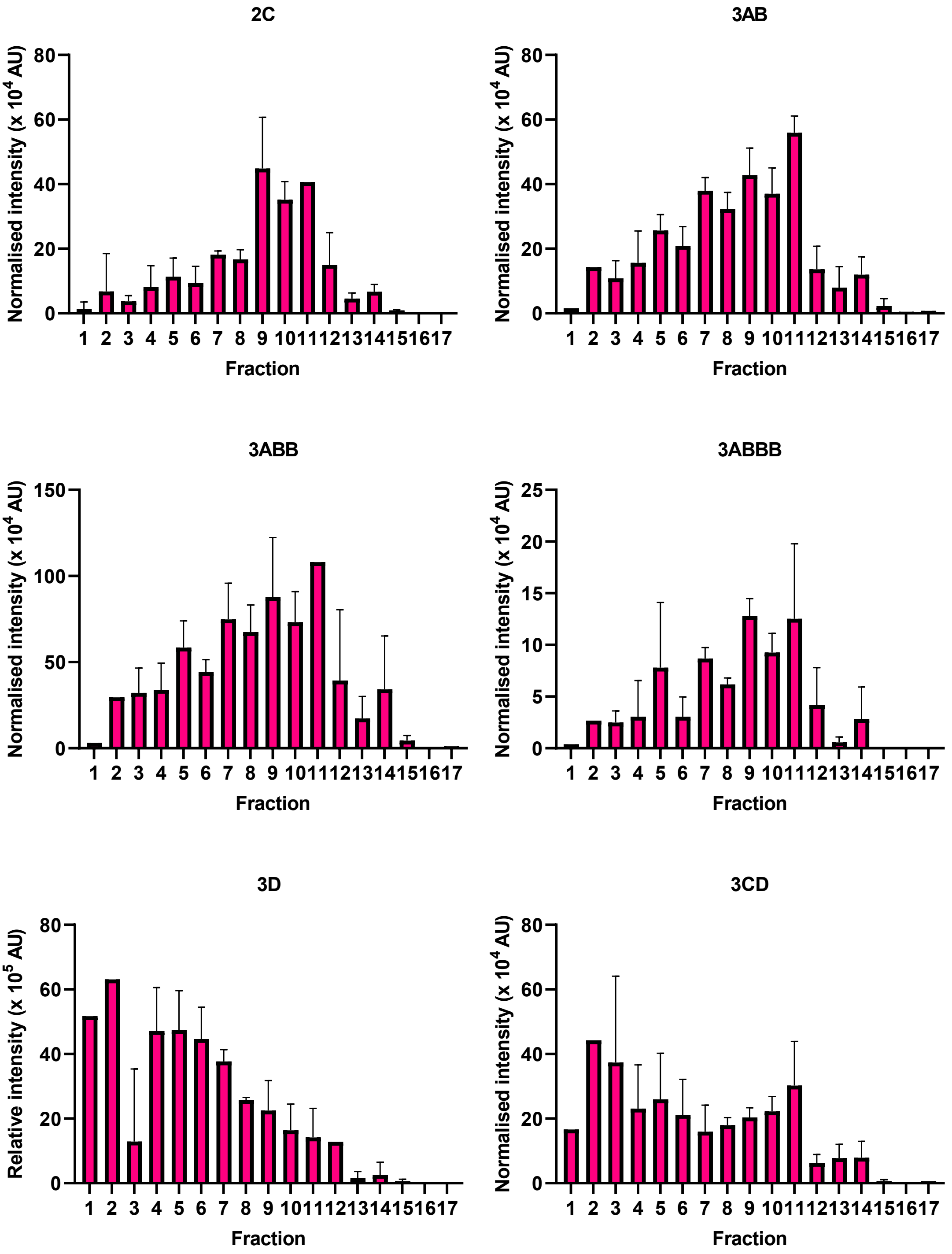

Figure S3. Densitometry of FMDV protein signal across primary gradient purification. Normalised to loading control of wild-type transfected lysate.

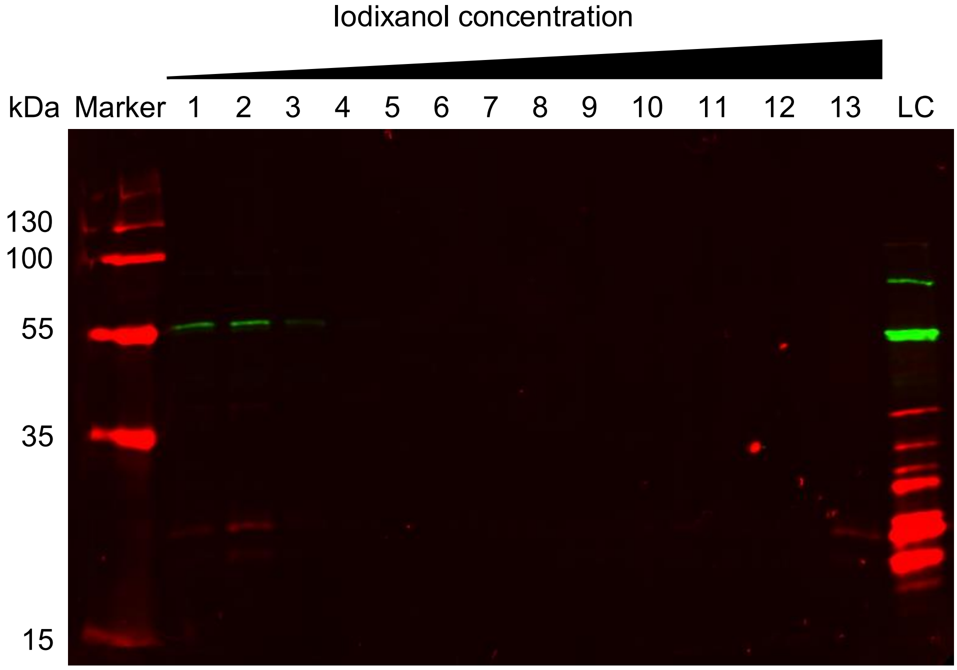

Figure S4. Primary gradient purification of GNN transfected lysate. Probed with anti-3D and anti-3A. LC = Loading control of wild-type transfected lysate.

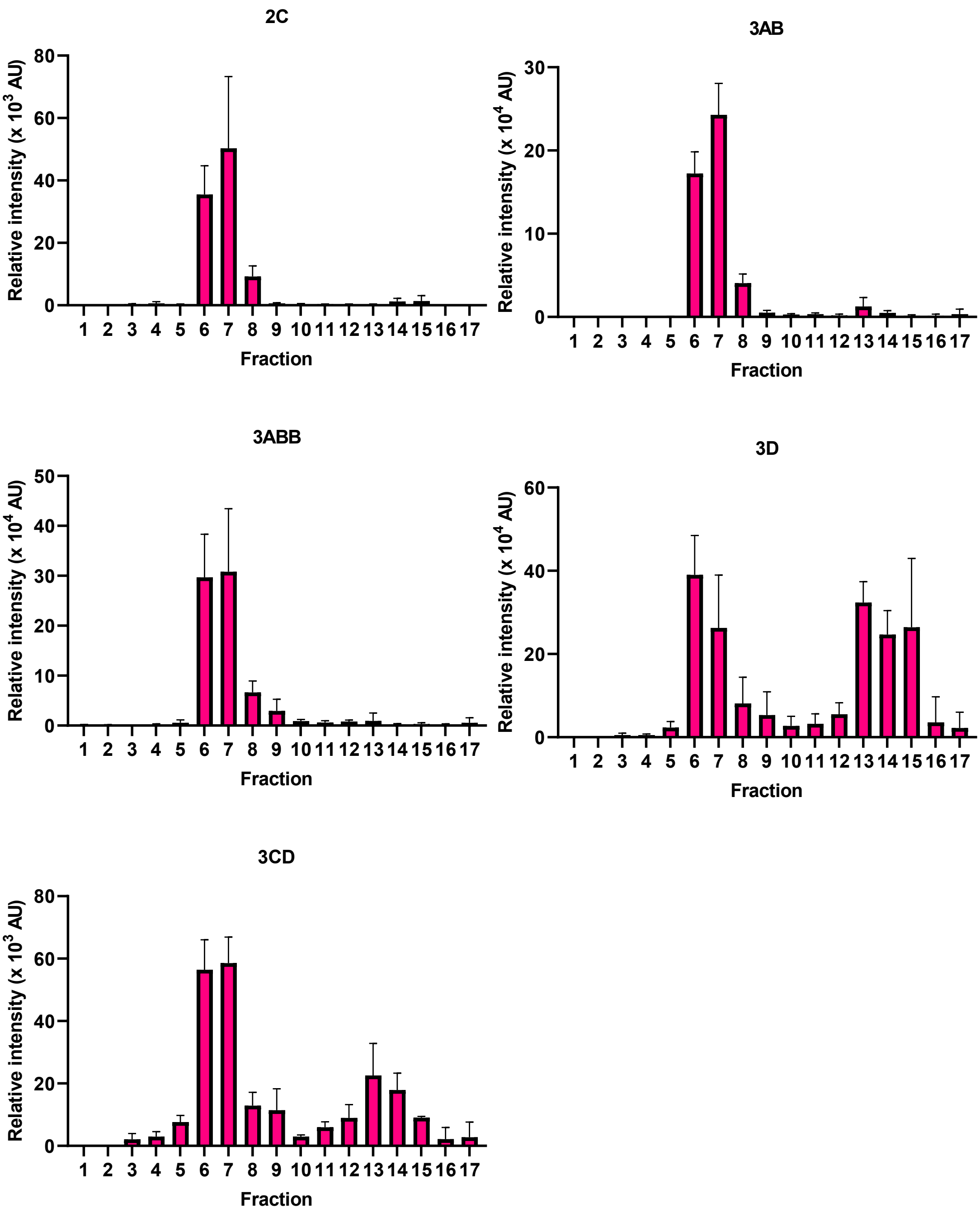

Figure S5. Densitometry of FMDV protein signal across secondary gradient purification.

Normalised to loading control of wild-type transfected lysate.

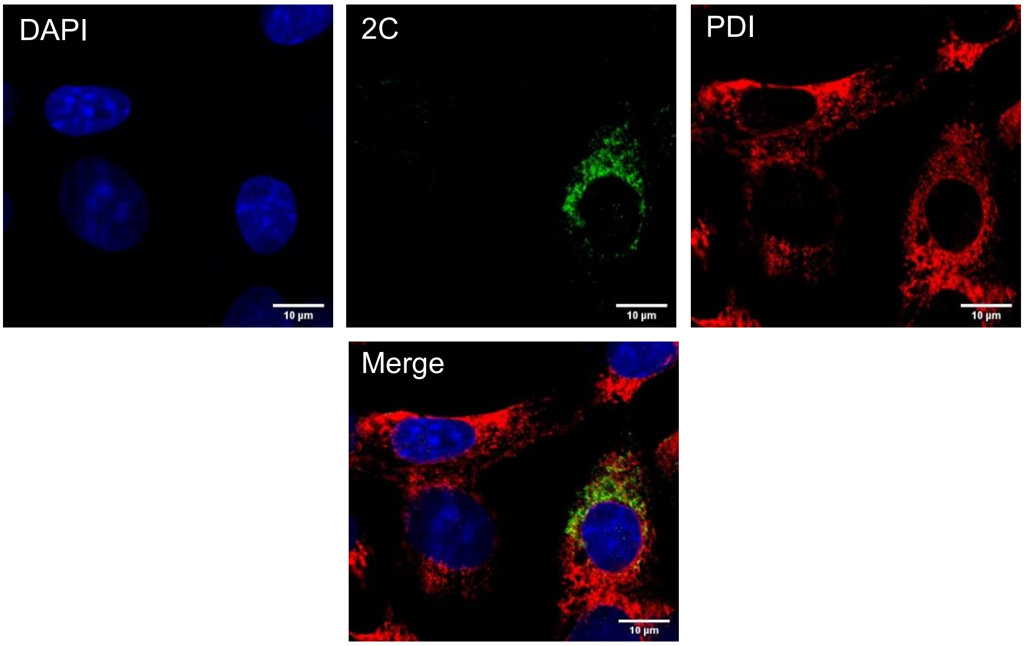

Figure S6. Immunofluorescence microscopy of FMDV 2C protein and an ER marker (PDI). BHK-21 cells were transfected with wild-type FMDV replicon before fixation at 2-hpt. Cells were subsequently immunostained with anti-2C (3-2) followed by anti-rabbit Alexafluor-555; and anti-PDI (1D3) followed by anti-mouse Alexafluor-647. Samples were stained with DAPI and visualised using a Zeiss LSM880 confocal microscope.

Fraction	GNN_n1	WT_n1	GNN_n2	WT_n2	GNN_n3	WT_n3
1	31	43	20	26	34	13
2	36	45	23	19	35	51
3	39	32	27	22	42	83
4	84	40	0	40	89	82
5	53	310	37	198	57	250
6	65	1252	45	603	67	1855
7	173	2755	114	1709	187	2611
8	226	1370	152	924	240	1543
9	309	404	204	401	324	850
10	320	491	214	383	340	403
11	450	1679	304	1094	444	1550
12	790	4133	531	2597	784	3960
13	2500	5061	1656	3718	2492	4505
14	2743	3506	1834	2427	2831	3204
15	550	766	364	862	2410	2100
16	2450	2765	3540	1950	2985	2840
17	1520	1920	1640	1489	1051	1400

Table S1. Raw ^{3}H scintiliation values from parallel secondary gradient purification and subsequent fractionation.
